# Integrated safety profile of atacicept: an analysis of pooled data from the atacicept clinical trial programme

**DOI:** 10.1093/rap/rkz021

**Published:** 2019-08-06

**Authors:** Caroline Gordon, Roberto Bassi, Peter Chang, Amy Kao, David Jayne, David Wofsy, Patricia Fleuranceau-Morel

**Affiliations:** 1 Rheumatology Research Group, Institute of Inflammation and Ageing, University of Birmingham, Birmingham, UK; 2 EMD Serono Research & Development Institute, Inc. (A Business of Merck KGaA, Darmstadt, Germany), Billerica, MA, USA; 3 Department of Medicine, University of Cambridge, Cambridge, UK; 4 Russell/Engleman Rheumatology Research Center, University of California, San Francisco, CA, USA

**Keywords:** atacicept, autoimmune diseases, safety, B-cell targeting, clinical trials, systemic lupus erythematosus, adverse events

## Abstract

**Objective:**

To characterize the overall safety profile of atacicept, we conducted an integrated analysis of pooled safety data from all 17 clinical studies to date.

**Methods:**

Three data sets were used to investigate safety endpoints: a double-blind placebo-controlled set (*n = *1568), an SLE set (*n = *761) and a full analysis set (*n = *1845; including all 17 studies).

**Results:**

Of 1568 patients in the double-blind placebo-controlled-set, 30.8% received placebo, and 8.2, 24.5 and 36.5% received atacicept 25, 75 and 150 mg, respectively. Treatment-emergent adverse event (TEAE) rates (adjusted by treatment-exposure) were generally higher with atacicept *vs* placebo, but no consistent association was found between atacicept dose and specific TEAEs or mortality. Serious infection and serious TEAE rates were similar for atacicept and placebo. The TEAE-related discontinuation rates were higher with atacicept *vs* placebo (16.1 *vs* 10.9/100 patient-years). In the full analysis set, 11 deaths occurred during treatment. Across indications, exposure-adjusted mortality rates/100 patient-years (95% CI) were 3.60 (0.90, 14.38), 0.34 (0.05, 2.43) and 1.18 (0.49, 2.82) with atacicept 25, 75 and 150 mg, respectively, and 0.44 (0.06, 3.12) with placebo. In SLE patients, exposure-adjusted mortality rates were 1.45 (0.54, 3.87) with atacicept 150 mg and 0.78 (0.29, 2.07) across all atacicept-treated patients. No deaths occurred with atacicept 75 mg or placebo. In the SLE and double-blind placebo-controlled sets, pharmacodynamic effects of atacicept were not associated with increased infection rates.

**Conclusion:**

The results of this integrated safety analysis support further development and evaluation of atacicept in selected patients for whom potential benefits might outweigh risks.

**Key message**

- Integrated analysis of atacicept clinical studies supports further evaluation in selected patients if benefits outweigh risks.

## Introduction

Atacicept is a fully human, soluble fusion protein consisting of a transmembrane activator and calcium modulating cyclophilin ligand (CAML) interactor extracellular ligand-binding domain and a modified Fc-IgG1 domain [1], which has been shown to bind and neutralize the cytokines B lymphocyte stimulator (BLyS) and a proliferation-inducing ligand (APRIL) *in vitro* [2]. BLyS and APRIL are key modulators of B-cell activity [3–6], and their levels have been shown to be elevated alongside dysregulated B-cell activity in various autoimmune conditions, including SLE [7, 8].

In *in vitro* studies and preclinical animal models, dual inhibition of BLyS and APRIL by atacicept was more potent than blocking of BLyS alone, resulting in decreased levels of autoreactive B cells, plasma cells and Ig [9–11]. Vigolo *et al.* [12] recently demonstrated that atacicept binding is not negatively affected by the loop region of the BLyS 60-mer (a naturally occurring cleaved human BLyS), which was shown temporarily to prevent binding of the anti-BLyS antibody, belimumab. Consistent with these data, atacicept reduces serum Ig levels in a dose-dependent manner in humans [13–15].

Atacicept has been investigated clinically in healthy volunteers [16, 17], patients with B-cell malignancies, such as chronic lymphocytic leukaemia, non-Hodgkin’s lymphoma, multiple myeloma and Waldenström’s macroglobulinaemia [18–20], and patients with autoimmune conditions, including RA [21–24], multiple sclerosis (MS) [25], LN [26], optic neuritis (ON) [27] and, most recently, SLE [14, 15, 28]; a study in IgA nephropathy is currently ongoing (NCT02808429).

Although the primary endpoints in two large SLE studies [APRIL-SLE (Phase II/III; NCT00624338) and ADDRESS II (Phase IIb; NCT01972568)] were not met, *post hoc* analyses suggested that weekly treatment with s.c. atacicept 150 mg had beneficial effects on disease activity and response rates, particularly in ADDRESS II, in patients with high disease activity (HDA; SLEDAI-2K ≥ 10) at screening [14, 15]. In APRIL-SLE, atacicept 150 mg reduced disease flare rates and prolonged the time to a new flare *vs* placebo [14]. However, two infection-related deaths in this group prompted early cessation of enrolment within the 150 mg dose arm. In ADDRESS II, a greater proportion of HDA patients treated with atacicept had SLE responder index [SRI]-4 and SRI-6 responses and a reduced risk for severe flare (as assessed by BILAG index A manifestation and by the Safety of oestrogens in Lupus Erythematosus National Assessment [SELENA]-SLEDAI flare index) compared with placebo-treated patients [15]. In both studies, the frequency of serious treatment-emergent adverse events (TEAEs; including infections) was comparable between atacicept and placebo groups [14, 15].

Safety findings, including unexpected decreases in IgG levels (APRIL-LN), serious infections (APRIL-LN and APRIL-SLE) including two cases of pneumonia with fatal outcome (APRIL-SLE), myocardial infarction with fatal outcome (Study 014 in LN) and increase of disease activity (ATAMS in MS and ATON in ON) [14, 25–27] were observed in earlier studies of atacicept in autoimmune diseases; these studies were partly (APRIL-SLE; atacicept 150 mg arm) or fully terminated as a consequence. After these observations, risk mitigation measures were implemented for the ADDRESS II study in SLE, its long-term extension and other subsequent studies. These measures included medical monitor reviews of patient screening data to confirm eligibility and up-to-date vaccinations against pneumococcus and seasonal influenza. It is worth noting that infection rates were lower in the ADDRESS II study and its extension than those observed in APRIL-SLE, and no study-drug-related deaths were reported [15, 28].

Given the observed benefit of atacicept, particularly in SLE patients with HDA, and the observed safety profile of atacicept in the ADDRESS II study and its long-term extension, there is rationale for further characterization of the overall safety profile of atacicept. This will serve as a foundation for future studies to explore the efficacy of atacicept further in specific subsets of patients, in whom the benefits might outweigh the potential risks. Therefore, we conducted an integrated analysis of safety data from all atacicept clinical studies to date to characterize the overall safety profile of atacicept. Specifically, we sought to investigate adverse event (AE) and infection rates with atacicept *vs* placebo in double-blind placebo-controlled (DBPC) trials, and mortality rates across all atacicept studies.

## Methods

### Studies included in the analysis

**Fig. 1:**
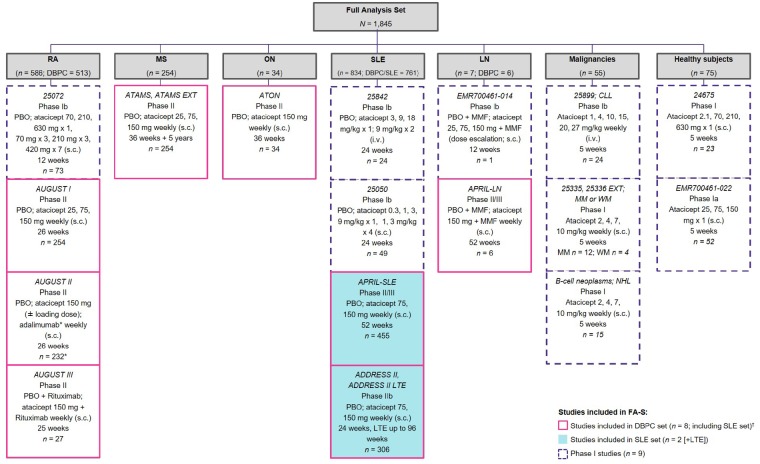
Study population *Patients treated with adalimumab (*n*=79) were not included in this analysis; ^†^Extensions were not included within the DBPC set. RA studies: 25 072 [24], AUGUST I [21], AUGUST II [22], AUGUST III [23]. MS studies: ATAMS and ATAMS EXT [25]. ON sudy: ATON [27]. SLE studies: 25 842 [30], 25 050 [29], APRIL-SLE [14], ADDRESS II and ADDRESS II LTE [15]. LN studies: EMR700461-014 (not published); APRIL-LN [26]. Studies in malignancies: 25 899 [19]; 25 335+25 336 EXT [20]; B-cell neoplasms [18]. Studies in healthy subjects: 24 675 [16, 17]; EMR700461-022 [17]. CLL: chronic lymphocytic leukaemia; DPBC: double-blind placebo-controlled; EXT: extension; LTE: long-term extension; MM: multiple myeloma; MS: multiple sclerosis; NHL: non-Hodgkin’s lymphoma; ON: optic neuritis; PBO: placebo; WM, Waldenström’s macroglobulinaemia.

Data from 17 clinical studies of atacicept were included in this integrated analysis (Fig. 1). Twelve studies were conducted in patients with autoimmune diseases (SLE [14, 15, 28–30], LN [26], RA [21–24], MS [25] and ON [27]), three studies focused on patients with B-cell malignancies (chronic lymphocytic leukaemia, non-Hodgkin’s lymphoma, multiple myeloma and Waldenström’s macroglobulinaemia) [18–20], and two were conducted in healthy volunteers [16, 17]. All studies have been described previously and are summarized in Supplementary Table S1, available at *Rheumatology Advances in Practice* online. Briefly, in the DBPC Phase II and Phase II/III studies, atacicept was administered s.c. weekly at doses of 25, 75 or 150 mg, and patients received concomitant standard-of-care therapies as appropriate. Study protocols were in accordance with the Declaration of Helsinki and were approved by the appropriate institutional review boards or ethics committees; written informed consent was provided by all patients.

### Data sets

Analyses were based on three separate pooled data sets to analyse all AEs (safety data) from all patients in the data set who were exposed to placebo or atacicept during the studies: (i) DBPC set, comprising safety data from eight atacicept Phase II or II/III DBPC studies conducted to date [not including long-term extension studies (LTEs)] [14, 15, 21–23, 25–27]; (ii) full analysis (FA) set, comprising safety data from all 17 clinical studies with atacicept, including those belonging to the DBPC set [14–30]; and (iii) SLE set, comprising safety data from the APRIL-SLE and ADDRESS II studies [14, 15, 28].

### Endpoints and assessments

Key safety endpoints analysed in the DBPC set included the overall incidence of AEs of special interest (AESI), TEAEs, serious AEs and TEAEs leading to treatment discontinuation. Pre-defined AESI categories were based on potential or theoretical risks and included infections, hypersensitivity and injection site reactions (ISRs), severe hypogammaglobulinaemia (IgG <3 g/l), cardiac events (cardiac arrhythmias, cardiac failure and ischaemic heart disorders), embolic and thromboembolic events, vestibular disorders, demyelinating disorders, malignant tumours, and depression and suicide ideation (Supplementary Appendix 1, available at *Rheumatology Advances in Practice* online). In the FA set, the overall number and causes of deaths and the exposure-adjusted mortality rates across the atacicept clinical trial programme were examined. In the SLE set, the association between changes from baseline in IgG levels and mature B-cell numbers and rates of serious and/or severe infection were assessed.

### Statistical analysis

Analyses were performed using descriptive statistics for continuous variables or by using the frequency count and percentage for categorical data. The TEAEs, AESIs and mortality rates were adjusted by atacicept exposure. The exposure-adjusted incidence rate (EAIR) in each treatment arm was defined as the ratio of the number of patients with an event to the sum of the duration of exposure to treatment of the patients up to the time of the first event or the end of observation (whichever occurred first) and was expressed as the rate per 100 patient-years. In patients with SLE, serious infections [defined by The International Council for Harmonisation of Technical Requirements for Pharmaceuticals for Human Use (ICH) as resulting in death or life-threatening] and/or severe infections [defined by the Common Terminology Criteria for Adverse Events as a significant impairment of function (Qualitative Toxicity Scale)] were analysed (as a combined term) per quartile of changes from baseline in the serum IgG levels and mature B-cell numbers.

## Results

### Study population (DBPC, FA and SLE sets)

The DBPC set included 1568 patients treated for a total of 841.4 patient-years (Table 1; SLE, 48.5%; LN, 0.4%; RA, 32.7%; MS, 16.2%; and ON, 2.2%). Of these, 483 patients (30.8%) received placebo, and 129 (8.2%), 384 (24.5%) and 572 (36.5%) received atacicept 25, 75 and 150 mg, respectively. Baseline demographics were balanced across treatment arms within the same indication (Table 1) [14, 15, 21–23, 25–28]. The FA set included safety data from 1845 patients, including healthy volunteers (*n = *75) [16, 17], patients enrolled in Phase I studies (B-cell malignancies, *n = *55; and autoimmune diseases, *n = *147) [18–20, 24, 29, 30] and patients in the LTEs of DBPC studies [25, 28]. The SLE set included 761 patients enrolled in Phase II/III studies; these patients were treated for a total of 652.4 patient-years. The treatment duration ranged from 24 to 52 weeks in the DBPC and SLE sets and from 4 to 260 weeks in the FA set (Supplementary Table S1, available at *Rheumatology Advances in Practice* online).

**Table 1:** Baseline demographics and disease characteristics by disease indication (double-blind placebo-controlled set)

	SLE		LN		RA		MS		ON	
	**Placebo** *n = *254	**Atacicept** *n = *507	**Placebo** *n = *2	**Atacicept** *n = *4	**Placebo** *n = *147	**Atacicept** *n = *366	**Placebo** *n = *63	**Atacicept** *n = *191	**Placebo** *n = *17	**Atacicept** *n = *17
Gender, *n* (%)										
Male	19 (7.5)	38 (7.5)	1 (50.0)	1 (25.0)	24 (16.3)	63 (17.2)	18 (28.6)	68 (35.6)	3 (17.6)	4 (23.5)
Female	235 (92.5)	469 (92.5)	1 (50.0)	3 (75.0)	123 (83.7)	303 (82.8)	45 (71.4)	123 (64.4)	14 (82.4)	13 (76.5)
Race, *n* (%)										
White	197 (77.6)	358 (70.6)	1 (50.0)	0 (0.0)	139 (94.6)	325 (89.0)	62 (98.4)	188 (98.4)	17 (100.0)	16 (94.1)
Black	8 (3.1)	23 (4.5)	1 (50.0)	3 (75.0)	0 (0.0)	13 (3.6)	1 (1.6)	2 (1.0)	0 (0.0)	0 (0.0)
Asian	36 (14.2)	89 (17.6)	0 (0.0)	1 (25.0)	1 (0.7)	11 (3.0)	0 (0.0)	0 (0.0)	0 (0.0)	0 (0.0)
Native American or Alaska Native	4 (1.6)	7 (1.4)	0 (0.0)	0 (0.0)	0 (0.0)	0 (0.0)	0 (0.0)	0 (0.0)	0 (0.0)	0 (0.0)
Other	9 (3.5)	30 (5.9)	0 (0.0)	0 (0.0)	7 (4.8)	16 (4.4)	0 (0.0)	1 (0.5)	0 (0.0)	1 (5.9)
Age, mean (s.d.), years	39.9 (12.5)	39.2 (11.9)	36.6 (25.9)	36.9 (11.3)	53.9 (11.3)	53.8 (11.9)	38.1 (10.5)	38.2 (9.7)	32.8 (7.1)	31.2 (10.2)
Weight, mean (s.d.), kg	68.8 (15.1)	68.5 (17.2)	88.7 (13.1)	82.9 (23.1)	74.2 (16.6)	74.4 (16.9)	69.2 (14.4)	72.6 (17.4)	71.2 (14.9)	68.3 (12.5)
Disease duration, mean (s.d.), years	5.9 (6.4)	6.4 (6.1)	1.6 (2.3)	1.1 (2.2)	10.3 (7.3)	10.6 (8.2)	4.4 (5.5)	4.1 (5.3)	0.03 (0.02)	0.02 (0.02)

MS: multiple sclerosis; ON: optic neuritis.

### Exposure-adjusted incidence of AESIs and TEAEs (DBPC set)

Treatment exposure was similar between the placebo and atacicept 75 and 150 mg groups (278.25, 225.02 and 286.67 patient-years, respectively), but was lower in the atacicept 25 mg group (51.48 patient-years) (Table 2). Treatment exposure was highest in patients with SLE and lowest in those with ON and LN (Supplementary Table S2, available at *Rheumatology Advances in Practice* online).

**Table 2:** Summary of exposure-adjusted incidence rate of adverse event of special interests and treatment-emergent adverse events by dose (double-blind placebo-controlled set)

	**Placebo** *n = *483	Atacicept				**All subjects** *n = *1568
		**25 mg** *n = *129	**75 mg** *n = *384	**150 mg** *n = *572	**All doses** *n = *1085	
Exposure, patient-years	278.25	51.48	225.02	286.67	563.16	841.41
Treatment-emergent AESIs, *n* (*n* per 100 patient-years)						
Infections	211 [107.78 (95% CI: 94.17, 123.35)]	43 [104.36 (95% CI: 77.40, 140.71)]	180 [118.67 (95% CI: 102.54, 137.33)]	281 [141.30 (95% CI: 125.71, 158.83)]	504 [128.65 (95% CI: 117.90, 140.39)]	715 [121.70 (95% CI: 113.10, 130.95)]
Non-opportunistic infections	211 (107.78)	43 (104.36)	180 (118.67)	281 (141.30)	504 (128.65)	715 (121.70)
Opportunistic infections	0 (0.0)	0 (0.0)	0 (0.0)	1 (0.35)	1 (0.18)	1 (0.12)
Herpes zoster infections	13 (4.73)	2 (3.92)	10 (4.50)	17 (6.06)	29 (5.24)	42 (5.07)
Severe infections	9 (3.24)	0 (0.0)	11 (4.94)	16 (5.59)	27 (4.82)	36 (4.29)
Serious^a^ infections	20 [7.26 [(95% CI: 4.68, 11.25)]	1 [1.94 (95% CI: 0.27, 13.79)]	23 [10.47 (95% CI: 6.96, 15.76)]	22 [7.71 (95% CI: 5.08, 11.71)]	46 (8.27 (95% CI: 6.19, 11.04)]	66 [7.93 (95% CI: 6.23, 10.10)]
Hypersensitivityb	37 (13.92)	8 (15.74)	40 (19.06)	55 (20.36)	103 (19.40)	140 (17.57)
Injection site reactions	54 (20.86)	27 (64.81)	109 (63.02)	156 (72.43)	292 (67.91)	346 (50.23)
Severe hypogammaglobulinaemia (IgG <3 g/l)	0 (0.0)	0 (0.0)	2 (0.89)	4 (1.40)	6 (1.07)	6 (0.71)
Cardiac arrhythmias^b^	18 (6.62)	11 (22.41)	23 (10.61)	25 (8.87)	59 (10.77)	77 (9.40)
Other arrhythmias	13 (4.75)	8 (16.18)	19 (8.69)	20 (7.07)	47 (8.53)	60 (7.28)
Atrial arrhythmias	0 (0.0)	3 (5.87)	3 (1.33)	3 (1.05)	9 (1.60)	9 (1.07)
Ventricular arrhythmias	5 (1.81)	0 (0.0)	4 (1.79)	6 (2.10)	10 (1.79)	15 (1.79)
Cardiac failure	6 (2.17)	2 (3.90)	7 (3.13)	15 (5.28)	24 (4.30)	30 (3.60)
Ischaemic disease and coronary artery heart disorders^b^	11 (3.99)	3 (5.91)	13 (5.92)	11 (3.87)	27 (4.87)	38 (4.57)
Embolic and thromboembolic events^b^	11 (3.96)	1 (1.95)	6 (2.67)	9 (3.16)	16 (2.85)	27 (3.22)
Vestibular disorders^b^	19 (7.01)	5 (9.90)	18 (8.30)	26 (9.26)	49 (8.94)	68 (8.30)
Demyelination^b^	1 (0.36)	1 (1.94)	0 (0.0)	5 (1.74)	6 (1.07)	7 (0.83)
Depression^b^	14 (5.08)	3 (5.83)	8 (3.58)	11 (3.87)	22 (3.93)	36 (4.31)
Malignant tumours^b^	0 (0.0)	1 (1.94)	1 (0.44)	3 (1.05)	5 (0.89)	5 (0.59)
TEAEs, *n* (*n* per 100 patient-years)						
Serious TEAE	51 (18.94)	15 (30.02)	51 (23.85)	61 (21.79)	127 (23.35)	178 (21.89)
Severe TEAE	28 (10.23)	10 (19.64)	45 (20.88)	56 (20.04)	111 (20.34)	139 (16.96)
Discontinuation due to TEAE	30 (10.85)	14 (27.57)	30 (13.39)	46 (16.13)	90 (16.07)	120 (14.34)

a Including some infections also classed as severe.

b Programmatically determined (crude results of the search) from a predefined list of MedDRA preferred terms according to the Standardized MedDRA Query (SMQ) or Customized MedDRA Query (CMQ) classification of the corresponding MedDRA version.

AESI: adverse event of special interest; TEAE: treatment-emergent adverse event.

Exposure-adjusted rates of AESI were assessed by treatment/dose (Table 2) and indication (Supplementary Table S2, available at *Rheumatology Advances in Practice* online). Infections were the most common treatment-emergent AESIs, and the EAIRs were generally higher with atacicept than with placebo (128.65 *vs* 107.78 per 100 patient-years). However, EAIRs of serious and severe infections were low across all patients (7.93 and 4.29 per 100 patient-years, respectively), with no notable differences between atacicept and placebo. Although the overall infection rates were highest with atacicept 150 mg, serious and severe infection rates were comparable to those with atacicept 75 mg. There was no notable increase in the rates of serious and severe infections with atacicept *vs* placebo in patients with SLE, RA or ON. In patients with LN or MS, the numbers of serious and severe infections were higher with atacicept than with placebo, but few patients presented with these events. Herpes zoster infections occurred infrequently overall [*n *=* *42 (5.07 per 100 patient-years)], and rates were similar between atacicept and placebo. These infections were most frequent in SLE patients (*n *=* *20) and occurred at slightly lower rates in RA and MS patients.

Hypersensitivity reactions occurred more frequently with atacicept *vs* placebo (19.40 *vs* 13.92 per 100 patient-years) but were mostly mild in severity. Hypersensitivity reactions were most frequent in patients with ON or LN and were similar among patients with MS, RA or SLE. One patient with SLE in the atacicept 75 mg group was hospitalized for anaphylactic shock after a bee sting; however, the causality was determined to be unrelated to atacicept. Rates of ISRs were higher with atacicept compared with placebo, with the highest incidence in the atacicept 150 mg group. However, ISRs were mostly of mild (*n = *117, 15.4%) or moderate (*n = *25, 3.3%) intensity, with three severe ISRs (0.4%). The EAIRs of ISRs were highest in patients with MS, LN and ON.

Severe hypogammaglobulinaemia (IgG <3 g/l) was infrequent across all treatment groups, occurring in two (0.5%) and four (0.7%) patients in the atacicept 75 and 150 mg groups, respectively, and being limited to patients with SLE (0.4%), LN (75.0%) or MS (0.5%). All patients with LN received loading doses of MMF (≥ 1 g/day) and high-dose CSs (prednisone, up to 60 mg/day) before the administration of atacicept.

The EAIRs of cardiac arrhythmias, cardiac failure and ischaemic heart disorders were higher with atacicept *vs* placebo. The highest incidence of cardiac arrhythmias was seen in the atacicept 25 mg group, and no evidence of an atacicept dose effect was observed. The highest EAIR of cardiac failure was observed in the atacicept 150 mg group (5.28 per 100 patient-years), and EAIRs of 3.90, 3.13 and 2.17 per 100 patient-years were observed with atacicept 25 and 75 mg and placebo, respectively. Analysis of these data by disease indication showed that cardiac arrhythmia rates were higher with atacicept than with placebo in patients with MS or RA, but were similar between atacicept and placebo in SLE patients. Ischaemic heart disorders were most common with atacicept 25 (5.91 per 100 patient-years) and 75 mg (5.92 per 100 patient-years) in the overall DBPC set and were more frequent with atacicept *vs* placebo in patients with RA (4.80 *vs* 1.62 per 100 patient-years). Embolic and thromboembolic events were more frequently reported in placebo-treated patients *vs* atacicept-treated patients (3.96 *vs* 2.85 per 100 patient-years), with the difference being most pronounced in patients with LN or MS.

The incidence of vestibular disorders was 8.94 *vs* 7.01 per 100 patient-years with atacicept (all doses) *vs* placebo, without evidence of an atacicept dose effect. Seven out of 1568 patients experienced a demyelination event, with a higher incidence being observed with atacicept than with placebo (1.07 *vs* 0.36 per 100 patient-years). Demyelination was observed only in patients with MS or ON and in a single patient with RA, for whom the demyelination event was unconfirmed.

Malignant or unspecified tumours were reported in a total of five (0.32%) atacicept-treated patients across the SLE (*n *=* *2), RA (*n *=* *2) and MS (*n *=* *1) groups, and no cases were observed with placebo; of note, the study sample included considerably more atacicept-treated than placebo-treated patients for these indications, and the overall exposure [sum of the duration of treatment of the patients (placebo or atacicept) until first AE or end of observation] was lower in the placebo group (Supplementary Table 2, available at *Rheumatology Advances in Practice* online).

The EAIRs of depression were higher with placebo than with atacicept (5.08 *vs* 3.93 per 100 patient-years); no cases of suicide ideation or suicidal behaviour were observed.

Serious TEAE rates were low overall and similar between atacicept and placebo groups. In atacicept-treated patients, the highest EAIRs were observed in the 25 mg group and the lowest in the 150 mg group (30.02 and 21.79 per 100 patient-years, respectively); however, these results should be viewed with caution owing to the smaller number of patients and lower exposure in the 25 mg group. Rates of severe TEAEs were higher with atacicept than with placebo but were similar across atacicept dose groups (Table 2). Analysis of TEAEs by disease indication showed that serious TEAE rates were higher with atacicept than with placebo only in patients with LN, RA and ON, and severe TEAEs were more commonly observed with atacicept than with placebo in all five indications. Discontinuation of treatment owing to TEAEs was relatively infrequent, but was more common with atacicept *vs* placebo (16.07 *vs* 10.85 per 100 patient-years) overall, with the highest rate being seen with atacicept 25 mg (27.57 per 100 patient-years) owing to the relatively smaller exposure time in this treatment arm (Table 2). Unadjusted discontinuation rates owing to TEAEs with atacicept were 10.9% with 25 mg, 7.8% with 75 mg and 8.0% with 150 mg. The TEAE-related discontinuations were most frequent in patients with LN and RA; in patients with SLE, there was no difference in discontinuation rates between atacicept and placebo.

### Most common TEAEs (unadjusted for exposure; DBPC set)

Infections and infestations were the most frequently reported TEAEs (45.6%), and frequencies were similar between atacicept 75 mg (46.9%), 150 mg (49.1%) and placebo (43.7%), but lower with 25 mg (33.3%; Table 3). The most commonly reported infections and infestations across all patients were urinary tract infections (10.2%), upper respiratory tract infections (10.1%), and nasopharyngitis (8.4%; Table 3; Supplementary Table S3, available at *Rheumatology Advances in Practice* online). The most frequently reported serious infection was pneumonia (atacicept 75 mg, *n *=* *9; atacicept 150 mg, *n *=* *8; and placebo, *n *=* *5).

**Table 3:** Summary of treatment-emergent adverse events ≥5% in any arm, by dose (double-blind placebo-controlled set)

	**Placebo** *n = *483	Atacicept				**All subjects** *n = *1568
System organ class Preferred term, *n* (%)		**25 mg** *n = *129	**75 mg** *n = *384	**150 mg** *n = *572	**All doses** *n = *1085	
Infections and infestations	211 (43.7)	43 (33.3)	180 (46.9)	281 (49.1)	504 (46.5)	715 (45.6)
Urinary tract infection	49 (10.1)	8 (6.2)	46 (12.0)	57 (10.0)	111 (10.2)	160 (10.2)
Upper respiratory tract infection	41 (8.5)	4 (3.1)	41 (10.7)	72 (12.6)	117 (10.8)	158 (10.1)
Nasopharyngitis	33 (6.8)	8 (6.2)	35 (9.1)	55 (9.6)	98 (9.0)	131 (8.4)
Bronchitis	19 (3.9)	4 (3.1)	22 (5.7)	39 (6.8)	65 (6.0)	84 (5.4)
General disorders and administration site conditions	100 (20.7)	42 (32.6)	145 (37.8)	201 (35.1)	388 (35.8)	488 (31.1)
Injection site reactions	39 (8.1)	24 (18.6)	83 (21.6)	117 (20.5)	224 (20.6)	263 (16.8)
Influenza-like illness	22 (4.6)	15 (11.6)	15 (3.9)	11 (1.9)	41 (3.8)	63 (4.0)
Injection site erythema	3 (0.6)	2 (1.6)	15 (3.9)	29 (5.1)	46 (4.2)	49 (3.1)
Gastrointestinal disorders	97 (20.1)	20 (15.5)	98 (25.5)	129 (22.6)	247 (22.8)	344 (21.9)
Diarrhoea	27 (5.6)	5 (3.9)	27 (7.0)	38 (6.6)	70 (6.5)	97 (6.2)
Nausea	14 (2.9)	8 (6.2)	25 (6.5)	26 (4.5)	59 (5.4)	73 (4.7)
Nervous system disorders	92 (19.0)	28 (21.7)	83 (21.6)	100 (17.5)	211 (19.4)	303 (19.3)
Headache	56 (11.6)	21 (16.3)	56 (14.6)	63 (11.0)	140 (12.9)	196 (12.5)
Musculoskeletal and connective tissue disorders	86 (17.8)	21 (16.3)	70 (18.2)	105 (18.4)	196 (18.1)	282 (18.0)
Back pain	27 (5.6)	1 (0.8)	24 (6.3)	20 (3.5)	45 (4.1)	72 (4.6)
Respiratory, thoracic and m ediastinal disorders	50 (10.4)	7 (5.4)	45 (11.7)	66 (11.5)	118 (10.9)	168 (10.7)
Cough	16 (3.3)	2 (1.6)	20 (5.2)	28 (4.9)	50 (4.6)	66 (4.2)

### Mortality across the atacicept trial programme (FA set)

Deaths that occurred across the atacicept clinical trial programme are listed in Table 4. Eleven deaths occurred during treatment, and exposure-adjusted mortality rates per 100 patient-years (95% CI) were 3.60 (0.90, 14.38), 0.34 (0.05, 2.43) and 1.18 (0.49, 2.82) with atacicept 25, 75 and 150 mg, respectively, and 0.44 (0.06, 3.12) with placebo (Table 5). One of the 11 deaths occurred after administration of a single dose of 25 mg atacicept; this case is described in Supplementary Appendix 2, available at *Rheumatology Advances in Practice* online. Two additional patients died after the completion of atacicept treatment: one patient with RA (9 months post-treatment; atacicept 210 mg) and one patient with SLE (18 months post-treatment; atacicept 150 mg). The exposure-adjusted mortality rate per 100 patient-years (95% CI) in patients with SLE was 0.78 (0.29, 2.07) across all atacicept-treated patients and 1.45 (0.54–3.87) with atacicept 150 mg; no deaths occurred with weight-based atacicept, atacicept 75 mg or placebo.

**Table 4:** Deaths registered across the atacicept programme (full analysis set)

Patient age and gender	Indication	Treatment dose	Event, days after first dose	Risk factors	Cause of death	Reference
Phase I, Ib and I/II studies						
60 years, male	RA	Atacicept 210 mg	286	Smoker	Lung cancer	Tak *et al.* (2008) [24]
80 years, male	CLL	Atacicept 2 mg/kg	41	Use of anti-coagulants (for atrial fibrillation); primary disease	Sepsis	Kofler *et al.* (2012) [19]
68 years, male	MM	Atacicept 7 mg/kg	74	Primary disease	Primary disease progression	Rossi *et al.* (2009) [20]
DBPC Phase II/III studies						
71 years, female	RA	Atacicept 25 mg	94	Vasculitis; CS use	Diverticular perforation	Genovese *et al.* (2011) [21]
70 years, female	RA	Atacicept 75 mg	149	Previous stroke; hypertension	Cardiorespiratory arrest	Genovese *et al.* (2011) [21]
67 years, male	RA	Atacicept 150 mg	55	History of cardiovascular disease; hypertension; diabetes	Sudden cardiac arrest	Van Vollenhoven *et al.* (2011) [22]
51 years, male	MS	Placebo	173	Chronic heart disease; coronary artery stenosis	Myocardial infarction	Kappos *et al.* (2014) [25]
22 years, male	SLE	Atacicept 150 mg	237	Digit ulcers secondary to scleroderma; endemic leptospirosis in country of residence	Acute respiratory failure; probable leptospirosis	Isenberg *et al.* (2015) [14]
30 years, female	SLE	Atacicept 150 mg	299	Primary disease; CS use	Pneumonia; pulmonary alveolar haemorrhage	Isenberg *et al.* (2015) [14]
61 years, female	SLE	Atacicept 150 mg	695	Advanced primary disease	Multi-organ failure	Isenberg *et al.* (2015) [14]
33 years, female	LN	Atacicept 25 mg	1	Smoker; hypercholesterolaemia; primary disease; chronic ischaemic heart disease	Acute myocardial infarction	see Supplementary Appendix 2, available at *Rheumatology Advances in Practice* online
32 years, female	SLE	Atacicept 150 mg	563	Primary disease; NSAID (COX2 inhibitor: meloxicam)	Thrombotic stroke	–
56 years, female	SLE	Atacicept 150 mg	368	NSAID (COX2 inhibitor: aceclofenac)	Unknown	–

CLL: chronic lymphocytic leukaemia; DBPC: double-blind placebo-controlled; MM: multiple myeloma; MS: multiple sclerosis.

**Table 5:** Exposure-adjusted mortality rates in patients treated with placebo or atacicept 25, 75 or 150 mg^a^

	**Placebo** *n = *431	**Atacicept 25 mg** *n = *130	**Atacicept 75 mg** *n = *384	**Atacicept 150 mg** *n = *677
Exposure, patient-years	227.68	55.63	291.66	425.26
Deaths, *n*	1	2	1	5
Exposure-adjusted mortality rate, per 100 patient-years (95% CI)	0.44 (0.06, 3.12)	3.60 (0.90, 14.38)	0.34 (0.05, 2.43)	1.18 (0.49, 2.82)

a Studies with single or multiple ascending doses of atacicept (*n *=* *111) and weight-based atacicept (*n *=* *112) were not included for this analysis (two deaths occurred in studies of weight-based atacicept).

Cardiac events were the most common cause of death in atacicept-treated patients (*n* = 4) and were deemed unrelated or unlikely to be related to treatment by the investigators.

### Infections in SLE patients (SLE set)

Unadjusted rates of serious and/or severe infections (combined term for analysis) and infestations in atacicept-treated patients were low and similar between the APRIL-SLE (19 of 295 patients; 6.4%) and ADDRESS II studies (including LTE; 13 of 206 patients; 6.3%). An analysis of serious and/or severe infections by quartile of serum IgG levels and changes in mature B-cell numbers from baseline showed no association between pharmacodynamic effects of atacicept and infection rates; these findings were also confirmed across the full DBPC set. Severe hypogammaglobulinaemia (IgG <3 g/l) occurred in two atacicept-treated patients with SLE (0.3% of all SLE patients; Supplementary Table S2, available at *Rheumatology Advances in Practice* online) and was not associated with the development of infection.

## Discussion

We conducted this integrated safety analysis of all 17 atacicept clinical studies to date, including eight DBPC studies, to characterize the overall safety profile of atacicept in patients with autoimmune diseases. Similar to observations with other biologic agents that are frequently used to treat autoimmune diseases [31], the most commonly observed AEs with atacicept were infections. This is not unexpected given the proposed B-cell-targeting mechanism of action of atacicept, which has been shown to reduce Ig levels and B- and plasma-cell numbers [13, 14, 22, 25–27]. Our observations are also consistent with findings from clinical studies of other BLyS-targeting therapies [32–34]. It should be noted that although there was an increase in overall infection rates with atacicept compared with placebo, the rates of serious and severe infections were not higher with atacicept in patients with SLE, RA or ON. Furthermore, although infection rates appeared to be increased with atacicept in patients with LN and MS, these observations should be viewed with caution given the overall low number of patients studied. Infection rates in patients with SLE enrolled in the ADDRESS II study were lower than those in patients enrolled in the APRIL-SLE study; this could be explained, in part, by the implementation of risk mitigation measures for ADDRESS II, following two pulmonary infections with fatal outcome in APRIL-SLE [14]. These mitigation measures, which included medical monitor reviews of patient screening data to confirm eligibility and up-to-date vaccinations against pneumococcus and seasonal influenza, have been and continue to be implemented in subsequent atacicept studies [15].

Given the immunogenic potential of any biological or biotechnology-derived protein, hypersensitivity reactions and ISRs were of special interest. As anticipated, exposure-adjusted hypersensitivity and ISRs were more frequent with atacicept than placebo. The highest frequencies were in patients treated with atacicept 150 mg, but most reactions were of mild to moderate intensity, with only a single case of anaphylaxis related to a bee sting, and no fatal reactions.

Hypogammaglobulinaemia was defined as another AE of particular interest, based on outcomes of the Phase II/III DBPC APRIL-LN study (NCT00573157), which was terminated early following an unexpected decline in IgG, together with several serious infections. However, analysis suggested that these events might have been attributable to factors other than atacicept treatment, because the reduction in serum IgG levels began 2 weeks before atacicept treatment, after the initiation or dose increase of MMF and CSs [26, 35]. This is consistent with a report by Broeders *et al.* [36], which showed that hypogammaglobulinaemia is common in renal transplant patients who are treated with MMF and CSs. The analysis of the pooled DBPC data in this report showed a low incidence of severe hypogammaglobulinaemia with atacicept (*n = *6 patients; 0.4%). Additionally, our analysis of serious and/or severe infections occurring in patients with SLE enrolled in the APRIL-SLE, ADDRESS II and ADDRESS II LTE studies showed no apparent association between reduced serum IgG and mature B-cell levels with serious and severe infection rates.

Although the incidence of malignancies was numerically higher with atacicept than with placebo, rates were overall low and appeared similar to the background rate of malignancies in SLE patients [37] and to rates reported with belimumab, based on 7 years of cumulative exposure data in SLE patients [38]. The analysed sample included considerably more atacicept-treated than placebo-treated patients, and the imbalance in patient numbers might therefore account for the numerical difference in malignancies.

Across all patients (FA set), the number of reported deaths was 13 (of 1845 patients in total), which is comparable to clinical studies of other BLyS- and/or APRIL-targeting agents [32, 33, 39]. Cardiac events were the most common cause of death across all atacicept studies (*n *=* *4 of 13 deaths) and were deemed unrelated or unlikely to be related to treatment. Analyses of the pooled DBPC data showed that EAIRs of cardiac events were low overall, with moderate increases for atacicept *vs* placebo observed only in patients with MS and RA. Despite evidence that patients with SLE are at an increased risk of cardiovascular disease [40], EAIRs of cardiac events observed in this analysis were lower in patients with SLE than in the other indications and were similar with atacicept and placebo. Interestingly, BLyS and APRIL are expressed in human arteriosclerotic plaques, suggesting that they could be a negative prognostic factor in cardiovascular disease [41].

### Implications for future studies in SLE patients

Long-term use of standard-of-care treatments for SLE, including CSs and immunosuppressants, is associated with adverse effects, including infection [42]; however, prolonged exposure to these agents is often unavoidable owing to the chronic nature of the disease [43]. Thus, there is a need for novel targeted therapies that not only provide greater efficacy, but also have a safety profile that is conducive to chronic use.

Although none of the atacicept studies to date has met its primary endpoint, clinical efficacy was demonstrated with atacicept 150 mg in two large Phase II/III SLE studies with different study designs (Supplementary Table S5, available at *Rheumatology Advances in Practice* online). In the APRIL-SLE study, atacicept 150 mg led to significant treatment benefits, with reduced flare rates and delayed time to the first flare over placebo (*post hoc* analysis of the discontinued arm) [14], and Gordon *et al.* [13] demonstrated that increased atacicept exposure associated with the 150 mg dose led to greater reductions in Ig levels and B-cell numbers (pharmacodynamic effects), without a significant increase in hypogammaglobulinemia or infection rates. In the ADDRESS II study, atacicept 150 mg showed significant treatment benefit over placebo in patients who had HDA at screening across different efficacy endpoints, and confirmed the consistent pharmacodynamic effects of atacicept treatment [15, 28, 44]. Furthermore, no clinically meaningful differences in safety data were observed between the modified intention-to-treat and the HDA populations in ADDRESS II [15]. Taken together, these findings support treatment of HDA patients with SLE with atacicept 150 mg in future studies [13–15].

The present analysis describes the EAIRs of most TEAEs in atacicept-treated patients. There was no consistent association with atacicept dose and cardiac arrhythmias, serious and severe infections, vestibular disorders, depression or malignant and unspecified tumours. By clarifying the frequency and severity of the potential risks associated with atacicept, this analysis provides a foundation for further investigation of the potential benefits of atacicept in SLE and other serious autoimmune diseases, while continuing to implement risk mitigation measures.

### Study limitations

Limitations of this integrated analysis include the differing designs and patient populations of the studies included. In addition, overall patient numbers varied considerably by disease and dose, and sample sizes were small in some indications. However, in the DBPC set, atacicept was investigated in 150–500 patients for each autoimmune indication (excluding ON and LN) and ∼130–570 patients for each dose.

### Conclusion

In conclusion, the outcomes of this integrated analysis of safety data from >1800 subjects support further development and evaluation of atacicept in selected patients for whom the potential benefits might outweigh the risks, with measures to minimize infection-related risks associated with B-cell-targeting therapies.

## Supplementary Material

rkz021_Supplementary_Data
