# Supplementary material for: Integrated safety profile of atacicept: an analysis of pooled data from the atacicept clinical trial programme
Source: Rheumatol Adv Pract. 2019 Aug 6;3(2):rkz021. doi: 10.1093/rap/rkz021 (PMC6735746; doi:10.1093/rap/rkz021)
Supplement: rkz021_Supplementary_Data [file rkz021_supplementary_data.docx]

# Supplementary Tables

## **Supplementary Table 1.** Summary of eligibility criteria and safety endpoints of all registered atacicept trials to date

| Study name or code (identifier) | Phase | Indication | Patients, *n* | | Treatment | | Duration, weeks | Eligibility | Safety Endpoints | Reference | |
| --- | --- | --- | --- | --- | --- | --- | --- | --- | --- | --- | --- |
| 25072 (n/a) | Ib | RA | 73 | PBO (*n*=18); atacicept 70 mg x 1 (*n*=6), 210 mg x1 (*n*=6), 630 mg x 1 (*n*=6); 70 mg x 3 (*n*=9), 210 mg x 3 (*n*=9), 420 mg x 7 (*n*=19) (s.c.). Repeated (x 3) doses were given at 2-week intervals | | 12 | | - Moderate-to-severe RA defined as ≥6 swollen joints, ≥6 tender joints, CRP ≥15 mg/L, or ESR ≥28 mm/hour - ≥6 months disease duration - Treatment failure with ≤5 DMARDs - RF positivity | - Physical examinations, vital signs, ECG, laboratory analysis, AEs and ISRs | Tak 2008 |  |
| 25842 (n/a) | Ib | SLE | 24 | PBO (*n*=4); atacicept 3 mg/kg x 1 (*n*=5), 9 mg/kg x 1 (*n*=5), 18 mg/kg x 1 (*n*=5), 9 mg/kg x 2 (*n*=5) given over 3 weeks (i.v.) | | 24 | | - 18–70 years with mild-to-moderate SLE (4/11 ACR criteria) - BMI <40 kg/m^2^ - SELENA–SLEDAI scores of 0–10 | - Physical examination including vital signs - ECGs - AEs - Local reactions including ISRs, pain (by VAS) - Laboratory analyses | Pena-Rossi 2009 |  |
| 25050 (n/a) | Ib | SLE | 49 | PBO x 1 (*n*=8); atacicept 0.3 mg/kg x 1 (*n*=6), 1 mg/kg x 1 (*n*=6), 3 mg/kg x 1 (*n*=6), 9 mg/kg x 1 (*n*=6), PBO x 4 (*n*=4), 1 mg/kg x 4 (*n*=7), 3 mg/kg x 4 (*n*=6) (s.c.). Repeated (x 4) doses were given weekly | | 24 | | - 18–24 years with mild-to-moderate SLE (4/11 ACR criteria) - BMI 18–40 kg/m^2^ - SELENA–SLEDAI) scores of 0–10 | - AEs, ECGs, physical examination, and haematologic and serum chemistry profiles evaluated using the Common Toxicity Criteria of the National Cancer Institute | Dall'Era 2007 |  |
| EMR700461-014 (NCT01369628) | Ib | LN | 1 | PBO + MMF, atacicept 25 mg, 75 mg, 150 mg + MMF (dose escalation; s.c.) | | 12 | | - Not published | - Not published | Not published* |  |
| 25899 (n/a) | Ib | CLL | 24 | Atacicept 1 mg/kg (*n*=3), 4 mg/kg (*n*=3), 10 mg/kg (*n*=3), 15 mg/kg (*n*=3), 20 mg/kg (*n*=3) ,27 mg/kg (*n*=6) weekly (i.v.) | | 5 | | - Refractory or relapsed CLL | - MTD, AEs, and DLTs - PK/PD | Kofler 2012 |  |
| 25335 and 25336 EXT (n/a) | I | MM/WM | 12 (MM) 4 (WM) | Atacicept 2 mg/kg (*n*=3), 4 mg/kg (*n*=3), 7 mg/kg (*n*=3), 10 mg/kg (*n*=7) weekly (s.c.) | | 5 | | - ≥18 years with relapsed and/or refractory MM or progressive WM - Measurable disease with ≥1 previous treatment - ≥6-month life expectancy - ECOG performance status 0–2 with adequate renal and hepatic functions - Stable haematological parameters | - AEs and DLTs - Physical examinations - Clinical laboratory tests | Rossi 2009 |  |
| B-cell Neoplasms (n/a) | I | NHL | 15 | Atacicept 2 mg/kg (*n*=4), 4 mg/kg (*n*=4), 7 mg/kg (*n*=4), 10 mg/kg (*n*=3) weekly (s.c.) | | 5 | | - ≥18 years with confirmed relapsed and/or refractory B-cell lymphoma - Measurable disease with high grade patients received a prior anthracycline-containing regimen - ≥6-month life expectancy - ECOG performance status 0–2 with adequate renal, hepatic and haematological functions | - DLTs (including AEs) - Hypersensitivity reactions | Ansell 2008 |  |
| 24675 (n/a) | I | Healthy volunteers | 23 | PBO (*n*=4); Atacicept 2.1 mg x 1 (*n*=5), 70 mg x 1 (*n*=5), 210 mg x 1 (*n*=5), 630 mg x 1 (*n*=4) (s.c.) | | 5 | | - 18–50 years, weight 60 kg and BMI 28 kg/m^2^ - Vital signs in normal range - Non-smokers ≥3 years | - AEs - Injection-site pain and local tolerability - Laboratory assessments | Munafo 2007 |  |
| EMR700461-022 (n/a) | Ia | Healthy volunteers | 52 | PBO (*n*=15); Atacicept 25 mg (*n*=13) x 1, 75 mg x 1 (*n*=12), 150 mg x 1 (*n*=12) (s.c.) | | 5 | | - 18–55 years - Body weight 45–90 kg (female) or 55–90 kg (male) - BMI of 18–29.9 kg/m^2^ | - TEAEs, SAEs - Physical examinations - AESI (ISR) and local tolerability - Pain | Willen 2015 |  |
| AUGUST I (NCT00430495) | II | RA | 254 | PBO (*n*=62); atacicept 25 mg (*n*=66), 75 mg (*n*=62), 150 mg (*n*=64) weekly (s.c.) | | 26 | | - 18 years with active RA defined as ≥8/66 swollen joints, ≥8/68 tender joints, and ≥10 mg/L CRP level ± ESR of ≥28 mm/hour - 1-year disease duration receiving ≥1 conventional DMARD - Previously inadequate response to TNFα antagonist therapy - Positive for RF and/or ACPA - Steroid treatment had to be at a stable dosage of ≤10 mg/day | - AEs - Clinical laboratory tests - Vaccine immunization status | Genovese 2011 |  |
| AUGUST II (NCT00595413) | II | RA | 232^†^ | PBO (*n*=76); atacicept 150 mg (± loading dose; *n*=78 for both groups) weekly; adalimumab^†^ 40 mg every other week (*n*=79) (s.c.) | | 26 | | - 18 years with active RA defined as ≥8/66 swollen joints, ≥8/68 tender joints, and ≥10 mg/L CRP level ± ESR of ≥28 mm/hour - 6 months disease duration - Previously inadequate response to MTX - Receipt of MTX stable dose for ≥28 says prior to study | - AEs - Local ISRs - Clinical laboratory tests | Van Vollenhoven 2011 |  |
| AUGUST III (NCT00664521) | II | RA | 27 | PBO + rituximab 1,000 mg (*n*=9); atacicept 150 mg + rituximab 1,000 mg (*n*=18) weekly (s.c.) | | 25 | | - 18 years with active RA - 1-year disease duration - DAS28 of >3.2 | - AEs - IgG level of < 3 g/L - Vital signs/safety laboratory parameters - Immunisation status | Van Vollenhoven 2015 |  |
| ATAMS (NCT00642902) and ATAMS EXT (NCT00853762) | II | MS | 254 | PBO (*n*=63); atacicept 25 mg (*n*=63), 75 mg (*n*=64), 150 mg (*n*=65) weekly (s.c.) | | 36 + 5 years | | - 18–60 years with RMS defined by the 2005 McDonald criteria - ≥2 relapses within 2 years and/or ≥1 relapse in 1 year and/or ≥1 gadolinium-enhancing lesion on T1-weighted MRI at screening | - AEs and infections - Laboratory abnormalities; ISRs; changes in vital signs; changes in ECG findings - Proportion of patients with antibodies to atacicept | Kappos 2014 |  |
| ATON (NCT00624468) | II | ON | 34 | PBO (*n*=17); atacicept 150 mg (*n*=17) weekly (s.c.) | | 36 | | - 18–60 years - Unilateral symptomatic ON as a first clinical demyelinating event (clinically isolated syndrome) and 28 days between the onset of symptoms and study day 1 | - AEs | Sergott 2015 |  |
| APRIL-SLE (NCT00624338) | II/III | SLE | 455 | PBO (*n*=157); atacicept 75 mg (*n*=159), 150 mg (*n*=145) weekly (s.c.) | | 52 | | - ≥16 years with mild-to-moderate SLE (4/11 ACR criteria) - Disease duration ≥6 months - Positive for ANA (HEp-2 ≥1:80) or dsDNA (≥30 IU/L) | - AEs - Clinical laboratory parameters, vital signs, ECGs and physical examinations - Changes in antibody titres to *S.* *pneumococcus*, tetanus toxoid and diphtheria | Isenberg 2015 |  |
| ADDRESS II (NCT01972568) and ADDRESS II LTE (NCT02070978) | IIb | SLE | 306 | PBO (*n*=100); atacicept 75 mg (*n*=102), 150 mg (*n*=104) weekly (s.c.) | | 24 up to 96 | | - ≥18 years with SLEDAI-2K ≥6 - 6-month disease duration - Positive for ANA (≥1:80) ± dsDNA (≥30 IU/ml) - Up-to-date vaccinations against *S. pneumoniae* and influenza virus | - AEs - Physical examination, vital signs, ECGs, clinical laboratory test | Merrill 2018; Wallace 2017 |  |
| APRIL-LN  (NCT00573157) | II/III | LN | 6 | PBO + MMF (*n*=2); atacicept 150 mg + MMF weekly (*n*=4) (s.c.) | | 52 | | - Active SLE (4/11 ACR criteria) - Positive ANA (≥ 1:80) and/or ds DNA ≥ 30 IU/ml - Active LN confirmed by renal biopsy - Haematuria (>10 RBC/hpf ±RBC cast) | - Ig levels - Infections | Ginzler 2012 |  |

*Outcomes of the EMR700461-014 study have not been published previously, but are described in this report (Supplementary Appendix 2).

^†^Patients treated with adalimumab (n=79) were not included in this analysis

AE: adverse event; AESI: adverse event of special interest; CLL: chronic lymphocytic leukaemia; DLT: dose-limiting toxicity; ECOG: Eastern Cooperative Oncology Group; EXT: extension; hpf: high power field; ISR: injection site reaction; i.v.: intravenous; LN: lupus nephritis; LTE: long-term extension; MM: multiple myeloma; MMF: mycophenolate mofetil; MS: multiple sclerosis; MTD: maximum tolerated dose; MTX: methotrexate; n/a: not available; NHL: Non-Hodgkin’s Lymphoma; ON: optic neuritis; PBO: placebo; PD: pharmacodynamic; PK: pharmacokinetic; RA: rheumatoid arthritis; RBC: red blood cell; RMS: relapsing multiple sclerosis; SAE: serious adverse event; s.c.: subcutaneous; SELENA, Safety of Estrogens in Lupus Erythematosus National Assessment; SLE: systemic lupus erythematosus; VAS: visual analogue scale; WM: Waldenstrom’s macroglobulinemia.

## **Supplementary Table 2.** Summary of exposure-adjusted incidence rates of AESI and TEAE, by disease (DBPC set)

|  | **SLE** | | **LN** | | **RA** | | **MS** | | **ON** | | **All placebo**  *n =* 483 | **All atacicept**  *n =* 1085 |
| --- | --- | --- | --- | --- | --- | --- | --- | --- | --- | --- | --- | --- |
|  | **Placebo**  *n =* 254 | **Atacicept**  *n =* 507 | **Placebo**  *n =* 2 | **Atacicept**  *n =* 4 | **Placebo**  *n =* 147 | **Atacicept**  *n =* 366 | **Placebo**  *n =* 63 | **Atacicept**  *n =* 191 | **Placebo**  *n =* 17 | **Atacicept**  *n =* 17 |  |  |
| Exposure, patient-years | 179.12 | 320.90 | 0.68 | 0.92 | 61.87 | 147.07 | 31.09 | 88.27 | 5.49 | 6.00 | 278.25 | 563.16 |
| **Treatment-emergent AESIs, *n* (*n* per 100 patient-years)** | | | | | | | | | | | | |
| Infections  Non-opportunistic  Opportunistic  Herpes zoster  Severe  Serious* | 131 (111.25)  131 (111.25)  0 (0.00)  8 (4.52)  6 (3.35)  18 (10.20) | 281 (139.81)  281 (139.81)  1 (0.31)  20 (6.39)  15 (4.71)  34 (10.82) | 0 (0.00)  0 (0.00)  0 (0.00)  0 (0.00)  0 (0.00)  0 (0.00) | 3 (340.30)  3 (340.30)  0 (0.00)  0 (0.00)  3 (336.12)  3 (336.12) | 48 (94.97)  48 (94.97)  0 (0.00)  4 (6.53)  2 (3.23)  1 (1.62) | 124 (104.20)  124 (104.20)  0 (0.00)  5 (3.41)  4 (2.72)  4 (2.72) | 28 (126.12)  28 (126.12)  0 (0.00)  1 (3.26)  0 (0.00)  0 (0.00) | 86 (130.18)  86 (130.18)  0 (0.00)  4 (4.58)  4 (4.54)  4 (4.54) | 4 (86.91)  4 (86.91)  0 (0.00)  0 (0.00)  1 (18.22)  1 (18.22) | 10 (207.29)  10 (207.29)  0 (0.00)  0 (0.00)  1 (16.66)  1 (16.66) | 211 (107.78)  211(107.78)  0 (0.00)  13 (4.73)  9 (3.24)  20 (7.26) | 504 (128.65)  504 (128.65)  1 (0.18)  29 (5.24)  27 (4.82)  46 (8.27) |
| Hypersensitivity^†^ | 24 (14.20) | 63 (21.26) | 1 (180.82) | 1 (108.71) | 6 (9.93) | 23 (16.15) | 4 (13.13) | 13 (15.05) | 2 (37.89) | 3 (61.94) | 37 (13.92) | 103 (19.40) |
| Injection Site Reactions | 29 (17.39) | 170 (72.46) | 1 (240.30) | 2 (1159.52) | 3 (4.92) | 13 (9.10) | 15 (55.91) | 92 (179.41) | 6 (154.44) | 15 (1295.21) | 54 (20.86) | 292 (67.91) |
| Severe hypogammaglobulinaemia (IgG<3 g/L) | 0 (0.00) | 2 (0.62) | 0 (0.00) | 3 (348.96) | 0 (0.00) | 0 (0.00) | 0 (0.00) | 1 (1.13) | 0 (0.00) | 0 (0.00) | 0 (0.00) | 6 (1.07) |
| Cardiac arrhythmias^†^  Atrial  Ventricular  Other | 11 (6.30)  0 (0.00)  2 (1.12)  9 (5.13) | 21 (6.70)  3 (0.94)  5 (1.57)  16 (5.08) | 1 (392.74)  0 (0.00)  0 (0.00)  1 (392.74) | 2 (303.11)  1 (108.71)  0 (0.00)  2 (303.11) | 1 (1.62)  0 (0.00)  0 (0.00)  1 (1.62) | 17 (11.82)  4 (2.73)  2 (1.36)  12 (8.30) | 2 (6.57)  0 (0.00)  1 (3.26)  1 (3.24) | 19 (22.71)  1 (1.14)  3 (3.43)  17 (20.19) | 3 (62.01)  0 (0.00)  2 (41.34)  1 (18.22) | 0 (0.00)  0 (0.00)  0 (0.00)  0 (0.00) | 18 (6.62)  0 (0.00)  5 (1.81)  13 (4.75) | 59 (10.77)  9 (1.60)  10 (1.79)  47 (8.53) |
| Cardiac failure | 3 (1.69) | 10 (3.14) | 0 (0.00) | 3 (680.59) | 1 (1.62) | 8 (5.49) | 1 (3.27) | 3 (3.41) | 1 (18.22) | 0 (0.00) | 6 (2.17) | 24 (4.30) |
| Ischaemic heart disorders & coronary artery disorders^†^ | 6 (3.37) | 13 (4.13) | 0 (0.00) | 1 (108.71) | 1 (1.62) | 7 (4.80) | 3 (9.87) | 5 (5.74) | 1 (19.33) | 1 (16.66) | 11 (3.99) | 27 (4.87) |
| Embolic & thromboembolic events^†^ | 6 (3.35) | 9 (2.82) | 0 (0.00) | 2 (525.54) | 2 (3.26) | 4 (2.73) | 3 (9.65) | 1 (1.13) | 0 (0.00) | 0 (0.00) | 11 (3.96) | 16 (2.85) |
| Vestibular disorders^†^ | 9 (5.15) | 22 (7.04) | 0 (0.00) | 0 (0.00) | 4 (6.61) | 11 (7.59) | 2 (6.59) | 14 (16.67) | 4 (88.44) | 2 (34.49) | 19 (7.01) | 49 (8.94) |
| Demyelination^†^ | 0 (0.00) | 0 (0.00) | 0 (0.00) | 0 (0.00) | 0 (0.00) | 1 (0.68) | 0 (0.00) | 3 (3.40) | 1 (19.46) | 2 (33.31) | 1 (0.36) | 6 (1.07) |
| Depression^†^ | 5 (2.81) | 8 (2.51) | 0 (0.00) | 1 (108.71) | 1 (1.62) | 3 (2.05) | 4 (12.98) | 7 (8.00) | 4 (83.72) | 3 (52.48) | 14 (5.08) | 22 (3.93) |
| Malignant tumours^†^ | 0 (0.00) | 2 (0.62) | 0 (0.00) | 0 (0.00) | 0 (0.00) | 2 (1.36) | 0 (0.00) | 1 (1.13) | 0 (0.00) | 0 (0.00) | 0 (0.00) | 5 (0.89) |
| **TEAEs, *n* (*n* per 100 patient-years)** | | | | | | | | | | | | |
| Serious TEAE | 39 (22.75) | 68 (22.24) | 0 (0.00) | 4 (1461.00) | 7 (11.54) | 39 (26.94) | 4 (12.87) | 14 (16.09) | 1 (18.22) | 2 (33.31) | 51 (18.94) | 127 (23.35) |
| Severe TEAE | 13 (7.37) | 44 (14.18) | 0 (0.00) | 4 (1718.82) | 7 (11.55) | 35 (24.38) | 4 (12.87) | 23 (26.79) | 4 (79.27) | 5 (86.55) | 28 (10.23) | 111 (20.34) |
| Discontinuation due to TEAE | 23 (12.94) | 41 (12.83) | 1 (152.19) | 3 (343.50) | 5 (8.11) | 40 (27.49) | 1 (3.22) | 6 (6.81) | 0 (0.00) | 0 (0.00) | 30 (10.85) | 90 (16.07) |

*****Including some infections also classed as severe; ^†^programmatically determined (crude results of the search) from a predefined list of MedDRA preferred terms according to the Standardized MedDRA Query (SMQ) or Customized MedDRA Query (CMQ) classification of the corresponding MedDRA version.

AESI: adverse event of special interest; DBPC: double-blind placebo-controlled; MedDRA: Medical Dictionary for Regulatory Activities; MS: multiple sclerosis; ON: optic neuritis; TEAE: treatment-emergent AE.

## **Supplementary Table 3.** Summary of Infections ≥2% in any arm, by dose (DBPC)

|  | **Placebo**  (*n =* 483) | **Atacicept** | | | | **All** **Subjects**  (*n =* 1568) |
| --- | --- | --- | --- | --- | --- | --- |
| MedDRA System Organ Class  Preferred Term |  | **25 mg**  (*n =* 129) | **75 mg**  (*n =* 384) | **150 mg**  (*n =* 572) | **Total**  (*n =* 1085) |  |
| Infections and Infestations, *n* (%) | 211 (43.7) | 43 (33.3) | 180 (46.9) | 281 (49.1) | 504 (46.5) | 715 (45.6) |
| Urinary tract infection | 49 (10.1) | 8 (6.2) | 46 (12.0) | 57 (10.0) | 111 (10.2) | 160 (10.2) |
| Upper respiratory tract infection | 41 (8.5) | 4 (3.1) | 41 (10.7) | 72 (12.6) | 117 (10.8) | 158 (10.1) |
| Nasopharyngitis | 33 (6.8) | 8 (6.2) | 35 (9.1) | 55 (9.6) | 98 (9.0) | 131 (8.4) |
| Bronchitis | 19 (3.9) | 4 (3.1) | 22 (5.7) | 39 (6.8) | 65 (6.0) | 84 (5.4) |
| Influenza | 19 (3.9) | 2 (1.6) | 9 (2.3) | 27 (4.7) | 38 (3.5) | 57 (3.6) |
| Sinusitis | 17 (3.5) | 2 (1.6) | 14 (3.6) | 17 (3.0) | 33 (3.0) | 50 (3.2) |
| Pharyngitis | 13 (2.7) | 0 (0.0) | 10 (2.6) | 16 (2.8) | 26 (2.4) | 39 (2.5) |
| Pneumonia | 6 (1.2) | 0 (0.0) | 12 (3.1) | 9 (1.6) | 21 (1.9) | 27 (1.7) |
| Gastroenteritis | 8 (1.7) | 1 (0.8) | 9 (2.3) | 8 (1.4) | 18 (1.7) | 26 (1.7) |
| Rhinitis | 3 (0.6) | 3 (2.3) | 9 (2.3) | 10 (1.7) | 22 (2.0) | 25 (1.6) |
| Cystitis | 3 (0.6) | 1 (0.8) | 8 (2.1) | 6 (1.0) | 15 (1.4) | 18 (1.1) |
| Tonsillitis | 2 (0.4) | 3 (2.3) | 4 (1.0) | 3 (0.5) | 10 (0.9) | 12 (0.8) |

DBPC: double-blind placebo-controlled; MedDRA: Medical Dictionary for Regulatory Activities.

## **Supplementary table 4.** Terminated atacicept studies*

| **Study** | **Phase** | **Indication** | **Reason for termination** |
| --- | --- | --- | --- |
| ATAMS (including its long-term extension) | II | MS | Increase in MS disease activity observed in patients in the ATAMS study |
| ATON | III | ON |  |
| Study 014 | IIb | LN | Sudden death of a patient from myocardial infarction after the first dose of atacicept 25 mg (see Supplementary Appendix 2) |
| APRIL-LN | II/III | LN | Decreased IgG levels were observed in 3 of 4 atacicept-treated patients (who were also receiving MMF), two of whom developed serious infections associated with severe hypogammaglobulinemia (IgG <3 g/L) |

*In the Phase II/III APRIL-SLE study the 150 mg atacicept treatment arm was discontinued following two deaths after episodes of pneumonia (not associated with hypogammaglobulinemia) but the overall trial was not terminated.
LN: lupus nephritis; MMF: mycophenolate mofetil; MS: multiple sclerosis; ON: optic neuritis.

## **Supplementary table 5.** Key efficacy outcomes in the APRIL-SLE and ADDRESS II studies

|  | Endpoint | Placebo | Atacicept 75 mg | Atacicept 150 mg |
| --- | --- | --- | --- | --- |
| **APRIL-SLE  (Isenberg D, et al. Ann Rheum Dis 2015)** | **Primary endpoint (ITT):** proportion of patients experiencing at least one BILAG A/B ﬂare over 52 weeks of treatment, flare rate in % (OR, [95% CI], p) | 54.0 | 58.0 (1.15 [0.73–1.80], p=0.543) | 37.0 (0.48 [0.30–0.77], p=0.002; post-hoc)* |
|  | **Key secondary endpoint (ITT):**  time to ﬁrst ﬂare during the 52-week treatment period, HR ([95% CI, p) | (reference) | 0.98 (0.69–1.40, p=0.929) | 0.56 (0.36–0.87, p=0.009; post-hoc)* |
| **ADDRESS II  (Merrill JT, et al. Arthritis Rheumatol 2018)** | **Primary endpoint (ITT):** proportion of patients with an SRI-4 response at 24 weeks, response rate in % (OR, [95% CI], p) | 44.0 | 57.8 (1.78 [1.01–3.12], p=0.045) | 53.8 (1.56 [0.89–2.72], p=0.121) |
|  | **HDA subpopulation:** proportion of patients with an SRI-4 response at 24 weeks, response rate in % (OR, [95% CI], p) | 42.3 | 60.0  (2.11 [0.97–4.59], p=0.060) | 62.7  (2.44 [1.09–5.44], p=0.029) |
|  | **HDA subpopulation:** proportion of patients with an SRI-6 response at 24 weeks, response rate in % (OR, [95% CI], p) | 28.8 | 43.6 (1.98 [0.88–4.46], p=0.098) | 54.9 (3.31 [1.44–7.61], p=0.005) |
|  |  | Placebo/atacicept 150 mg (switch after 24 weeks) | Atacicept 75 mg | Atacicept 150 mg |
| **ADDRESS II LTE  (Wallace DJ, et al. EULAR 2017; Morand E, et al. EULAR 2018)** | **ITT:** Proportion of patients with an SRI-4 response at 48 weeks, response rate in % | 48.0 | 55.9 | 58.7 |
|  | **HDA subpopulation:** Proportion of patients with an SRI-6 response at 48 weeks, response rate in % | 42.3 | 54.5 | 60.8** |
|  | **HDA subpopulation:** Patients with LDA at Week 48, attainment rate in % (OR, [95% CI]) | 19.2 | 21.8  (1.17, [0.46–3.00]) | 39.2 (2.71, [1.11–6.60])** |

*Post-hoc analysis following the discontinuation of the treatment arm due to two deaths.

**p<0.05 vs placebo/atacicept 150 mg; subjects on placebo group during the double-blind placebo controlled treatment period were switched to atacicept 150 mg at Week 24 for the long-term extension study.

CI: confidence interval, HDA: high disease activity, HR: hazard ratio, ITT: intention-to-treat, LDA: low disease activity, OR: odds ratio, SRI: SLE responder index

# Supplementary Appendix 1. Adverse events of special interest

Adverse events of special interest were:

- Infections
  - Serious infections
  - Severe infections
  - Non-opportunistic infections
  - Opportunistic infections
  - Herpes Zoster
- Hypersensitivity reactions (cases reviewed in line with Simpson criteria for anaphylaxis)
  - Anaphylactic reaction
  - Anaphylactoid Shock
  - Angioedema
  - Asthma Bronchospasm
  - Other hypersensitivity reaction (by a pre-specified list of preferred terms)
- Injection site reactions
- Severe hypogammaglobulinaemia
  - Defined as IgG<3 g/L
- Cardiac events
  - Cardiac arrhythmias (atrial, ventricular and atrial/ventricular)
  - Cardiac failure
  - Ischaemic heart disease and coronary artery disorders
- Embolic and thrombotic events
- Vestibular disorders
- Demyelination
- Depression and suicide
  - Depression
  - Suicide ideation
- Tumours
  - Malignant
  - Unspecified

# Supplementary Appendix 2. Phase Ib LN Study Patient Case Details

We describe here the outcome of the Phase Ib dose-escalation EMR700461-014 study designed to examine the safety and tolerability of atacicept in patients with LN receiving a stable regimen of mycophenolate mofetil (MMF) or sodium (MPS) formulation, with glucocorticoids.

Enrolment of up to 35 patients with LN was planned, but following the death of the first patient and before the microscopic autopsy report became available and the cause of death defined, the trial was prematurely terminated.

The recently diagnosed (5 years) patient with SLE was a 33-year-old woman, who developed LN approximately 19 months before she was enrolled into the study, when a renal biopsy revealed a mixed proliferative class IV (A/C) and membranous (class V) glomerulonephritis. Her relevant medical history included psoriasis, mixed connective tissue disease, SLE, lupus glomerulonephritis, and hypercholesterolemia. The patient was a cigarette smoker (20 cigarettes/day) with no reported history of hypertension or diabetes.

At screening, the patient was receiving prednisone (5 mg/day), MMF (1000 mg twice daily), olmesartan (20 mg/day), atorvastatin (20 mg/day), and Calcium Sandoz® Forte + D (Novartis; 2 tablets/day). Vital signs, cardiac and respiratory examinations, chest X-ray and electrocardiogram were within normal limits.

On the day of enrolment, laboratory results showed that the parameters tested were in the normal range (Table).

The patient received a single dose of atacicept 25 mg, administered subcutaneously, at 10.40 am; and left the study visit at midday experiencing mild fatigue, which had resolved by the evening. Early the following morning, the patient awoke with neck pain, which she had been feeling repeatedly in the weeks preceding the screening visit, and vomited after taking ibuprofen. No fever, visual changes, swelling, rash, or itching were reported but her husband later found that she was cold and was not breathing. Resuscitation efforts by her husband and emergency services were unsuccessful.

The macroscopic autopsy report indicated severe intimal hypertrophy in the coronary arteries, most prominently in the anterior descending coronary artery (~90%), and atheroma lesions without significant obstruction and no sign of stroke. Massive pericardial adhesions, mainly in the left ventricle, were noted. Pulmonary and hepatic congestion were also noted. The report listed acute myocardial infarction as the cause of death, but highlighted the requirement for microscopic confirmation.

The final autopsy report, which was provided approximately 6 months after the event, indicated “moderate coronary atherosclerosis with acute wall thrombosis in the anterior descending artery compatible with acute coronary syndrome; ischaemic myocardial foci, of at least a number of weeks’ duration, in various ventricular aspects, with predominance in the anterior aspect of the left ventricle; and mild acute myocardial ischaemia of the apex.” Based on the final report, the investigator determined that the death was unrelated to atacicept treatment.

The report concluded “determination of the cause of death: acute heart failure secondary to acute coronary syndrome: acute mural thrombosis of the anterior descending artery.”

Table. Phase Ib LN study patient laboratory values at enrolment

| Parameter | Result | Normal range |
| --- | --- | --- |
| Anti-DNA antibodies | 11 IU/mL | 0–6.3 IU/mL |
| Anti-cardiolipin antibodies (IgG) | Negative |  |
| Anti-cardiolipin antibodies (IgM) | Negative |  |
| Anti-La antibodies | Negative |  |
| Anti-Ro antibodies | Negative |  |
| Anti-Smith antibodies | Positive |  |
| Anti-nuclear antibodies | Positive |  |
| Activated partial thromboplastin time | 24.5 seconds | 24.3–30.4 seconds |
| Prothrombin time INR | 1.1 | 0.8–1.2 |
| Lupus anticoagulant | 1.02 | 0–1.37 |
| Serum IgG | 9.44 g/L | 7–16 g/L |
| 24-hour urinary protein | 4.50 g/24 hours | 0.04–0.15 g/24 hours |
| Urine protein/creatinine | 505.3 mg/mmol | 0–22.6 mg/mmol |
| Serum albumin | 35 g/L | 32−55 g/L |

INR: international normalized ratio.
